# Experience of Patients with Diabetes and Other Cardiovascular Risk Factors with Health Professionals and Healthcare in Spain

**DOI:** 10.3390/jcm10132831

**Published:** 2021-06-26

**Authors:** Domingo Orozco-Beltrán, Sergio Cinza-Sanjurjo, José Escribano-Serrano, Flora López-Simarro, Gonzalo Fernández, Antón Gómez-García, Marta Cedenilla-Horcajuelo, Karine Ferreira de Campos

**Affiliations:** 1 Department of Clinical Medicine, Miguel Hernandez University, 03202 Elche, Spain; dorozcobeltran@gmail.com; 2 Healthcare Center Porto do Son, Healthcare Area of Santiago de Compostela, Research Institute Santiago de Compostela (IDIS), Centro de Investigación Biomédica en Red–Cardiovascular Diseases (CIBERCV), 15970 La Coruña, Spain; scinzas@semergen.es; 3 Healthcare Center San Roque, 11360 Cádiz, Spain; jescribano19@gmail.com; 4 Basic Health Area Martorell Urbano, Institut Català de la Salut, 08760 Barcelona, Spain; floralopezsimarro@gmail.com; 5 Medical Affairs Department, MSD Spain, 28027 Madrid, Spain; gonzalo.fernandez@merck.com (G.F.); anton.gomez@merck.com (A.G.-G.); marta.cedenilla@merck.com (M.C.-H.)

**Keywords:** type 2 diabetes mellitus, patient experience, healthcare, new technologies in primary healthcare

## Abstract

We aimed to evaluate the experience of patients with type 2 diabetes (T2DM) with healthcare received in Spain. This was a retrospective, observational study in patients with T2DM cared for in primary healthcare (PHC) centers. A cross-sectional analysis of the patients’ experience data was performed using the Instrument for the Evaluation of the Experience of Chronic Patients (IEXPAC). A total of 475 patients with T2DM were recruited from 36 PHC centers, of which 248 (52.2%) completed the IEXPAC questionnaire. The IEXPAC total mean score (range 0–10) was 7 points, with an average “new relational model” score of 2.5 points. The mean continuity of care score after hospital discharge was 6.2 points. The results showed that 8% of the patients always or almost always used the internet to check their medical history, appointments or other data from their healthcare service, and 15% responded that healthcare professionals always or almost always informed them of forums or other reliable internet sites to obtain information about their illness. The study results show that there is a wide margin for improvement in the experience of patients with T2DM with healthcare in Spain, especially regarding the information patients receive or can obtain.

## 1. Introduction

The presence of multiple chronic diseases in the same patient (so-called multimorbidity) has a significant impact on the physical, mental and social well-being of the patient [1,2,3]. In addition, multimorbidity increases the complexity of healthcare [4], resulting in a worse quality of care [5].

In Spain, diabetes has an overall prevalence of 13.8% [6], reaching 19% in primary care centers [7]. In addition, the prevalence of diabetes seems to be constantly growing in our environment [8], and diabetes is, along with hypertension, one of the most multimorbid chronic diseases [9]. An analysis of a Spanish database with a total of 373,185 patients with type 2 diabetes mellitus (T2DM) showed that 82% of patients exhibited ≥2 comorbidities and 31% exhibited ≥4 comorbidities [10]. Similar results have been reported in other studies in our setting [11].

In 1996, Wagner et al. [12] proposed the “Chronic Care Model”, a framework for the care of patients with chronic diseases that could improve the quality of healthcare of these patients. This model represented a systematic transformation of healthcare systems to provide proactive, integrated, and patient-centered care. From his observations, today’s care is considered to be of quality when it is effective in maintaining or improving health and is patient centered, i.e., it respects and responds to individual needs, preferences and values [13]. Patient experience, along with efficiency and safety, has become one of the three key components of healthcare, and it is interrelated with these components [14]. Thus, the patient experience is positively associated with health outcomes, adherence to medical recommendations, preventive care, the use of health resources and adverse events [14]. Therefore, the information provided by patients can be used for initiatives that improve the quality of care and services [15], and it has become a key indicator of healthcare [16].

A study was conducted in Spain to evaluate patients’ experiences with healthcare in four chronic diseases: rheumatic diseases, inflammatory bowel disease, human immunodeficiency virus (HIV) infection and diabetes mellitus [17]. The study, using the “Instrument for the Evaluation of the Experience of Chronic Patients” (IEXPAC) questionnaire, showed some differences among diseases in terms of patient experience, and it also showed areas of improvement in overall healthcare [17]. However, with regard to diabetes, we only have global data reported in the primary publication [17].

We present herein the results of the experience of diabetic patients with healthcare services in a study initially intended to evaluate the prevalence of good adherence to antidiabetic, antihypertensive and lipid-lowering medication in patients with type 2 diabetes and the presence of comorbidities such as hypertension and dyslipidemia.

## 2. Materials and Methods

### 2.1. Study Design and Study Population

This was a retrospective, observational study conducted by 80 primary care physicians who had to recruit five consecutive patients during a single study-inclusion visit; the results shown are those relating to a cross-sectional analysis. The study was approved by the Ethics Committee of each participating center, and informed consent was obtained from all participants.

Patients included were at least 18 years of age, were diagnosed with type 2 diabetes, had received oral antidiabetics and antihypertensive drugs for the treatment of high blood pressure and lipid-lowering agents for the treatment of dyslipidemia for at least 12 months prior to inclusion and were monitored by their primary care physician. Patients were excluded if they were dependent, had participated in any clinical trial in the year prior to inclusion, had a psychiatric disorder other than depression or anxiety, had a serious or terminal illness, were receiving insulin or some glucagon-like peptide-1 (GLP-1) analog or had been pregnant or diagnosed with diabetic ketoacidosis, diabetes-related malnutrition, drug-induced diabetes or gestational diabetes in the year prior to inclusion.

### 2.2. Assessments

Demographic and clinical data were obtained from the electronic medical history and, if not available, collected during the inclusion visit. In addition to demographic data, information from physical examination, diabetes-related complications and the presence of comorbidities other than hypertension and dyslipidemia were collected. Information on the degree of glycemic control, hypertension or dyslipidemia was collected in addition to the last measurement of glycated haemoglobin (HbA1c), Low-density lipoprotein cholesterol (LDLc) and blood pressure (BP) within the last 3 months; if needed, these data were obtained at the time of the inclusion visit. Information on the medication received through the e-prescription system was also obtained.

The patient was requested to complete the IEXPAC questionnaire in private, without the influence of any healthcare professional, and return it to the investigator in a closed envelope. The IEXPAC scale used in this study was the 2015 version containing 11 + 1 items [18]. All items referred to the previous 6 months, except for the hospitalization item that referred to the previous 3 years. The first 11 items evaluated the various patient interactions with professionals and healthcare services on a frequency Likert-type scale with the following response alternatives and scores: always (5 points), almost always (4 points), sometimes (3 points), almost never (2 points) and never (1 point). Thus, the sum of the scores of the 11 IEXPAC items gives a direct score ranging from 11 to 55 points, which transforms into a scale of 0 to 10 using the following formula:

***Global score IEXPAC* =** 10 × (*sum of scores of* 11 *items* − 11)/44.

The score of item 12, regarding continuity of care after hospital discharge, was communicated separately and converted to a 0–10 scale, with the following alternatives of response and scores: always (10 points), almost always (7.5 points), sometimes (5 points), almost never (2.5 points) and never (0 points). The questionnaire also had 3 dimensions: 1. Productive interactions (items 1, 2, 5 and 9): type, content and intensity of interactions between patients and professionals aimed at achieving better results; 2. New relational model (items 3, 7 and 11): new forms of patient interaction: non-in-person methods, such as internet or interactions with other patients; and 3. Patient self-management (items 4, 6, 8 and 10): the person’s ability to manage his/her care and improve his/her well-being owing to the actions carried out by professionals. The last item asked about follow-up after hospital discharge, if required. Any score below 10 (i.e., there is “always” good care) points to a margin of improvement in the health organization in terms of care for its chronic patients [18].

### 2.3. Statistical Analysis

Medication adherence was calculated as the proportion of days covered (PDC) by medication. The average PDC was calculated for all drugs within a category. Patients were considered adherents in a particular category if the average was 80% or higher. A patient was considered globally adherent if the PDC was ≥80% for each of the three drug categories.

Glycemic control was assessed following the recommendations of clinical practice guidelines; thus, it was considered controlled if HbA1c < 7%. High blood pressure was considered controlled when systolic blood pressure was <140 mmHg and diastolic BP was <90 mmHg. Finally, patients were considered controlled with respect to hyperlipidemia if LDLc was <100 mg/dL in the case of primary prevention and <70 mg/dL for secondary prevention.

Quantitative variables are described as the mean and standard deviation, and qualitative variables are described as absolute and relative frequencies. No imputation of missing values was performed. If any IEXPAC items were lost in any patient, that patient was not included in the total score or the corresponding dimension score. An unpaired Student’s test was used to compare means. All tests were two-sided and considered significant if *p* < 0.05. The analyses were performed with the SPSS 20.0.0 statistical package (IBM Corp., Chicago, IL, USA).

## 3. Results

### 3.1. Patient Location and Characteristics

Thirty-six centers in four autonomous communities (Galicia, Andalusia, Catalonia and Valencia) recruited 475 patients between May and November 2016. Of these, 248 (52.2%) returned the completed IEXPAC questionnaire, and 229 provided a questionnaire with no missing data and contributed to the IEXPAC total score. Out of the total number of patients who completed the IEXPAC questionnaire, 124 answered the hospitalization item.

Patients were of an advanced age (mean (standard deviation), 70.9 (9.2) years), with a balanced distribution of men and women and a relatively high frequency of diabetes-related complications (Table 1). Patients were polymedicated with a mean of close to 10 drugs per patient.

### 3.2. Degree of Control of Cardiovascular Risk Factors and Adherence

Regarding the degree of control, 175 (70.6%) patients had HbA1c values below 7.0%; 161 (64.9%) had blood pressure below 140/90; 158 out of 245 evaluable patients (64.5%) had LDLc below 100 mg/dL or 70 mg/dL; and 76 out of 245 evaluable patients (31.0%) had three risk factors controlled according to the aforementioned criteria.

Regarding adherence, 211 (85.1%) were compliant with their oral antidiabetics; 232 (93.5%) were compliant with their antihypertensive drugs; 213 (85.9%) took their lipid-lowering medication; and 183 (73.4%) were compliant with all three drug groups.

### 3.3. Patient Experience with Healthcare Professionals and Services

**Figure 1:**
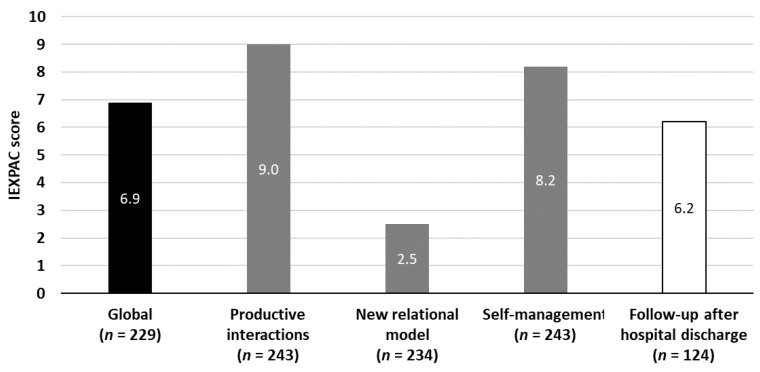
Patient experience with healthcare professionals and services: IEXPAC total scores. IEXPAC (the Spanish abbreviation for “The Instrument for the Evaluation of the Experience of Chronic Patients”). A total of 248 patients provided information about IEXPAC; 19 had missing data on some items and were therefore not included in the total score.

The IEXPAC total mean score was 6.9 points, greatly influenced by the “new relational model” dimension score, with a mean of 2.5 points (Figure 1). The follow-up score after hospital discharge was also low, with a mean of 6.2 points.

When scores were analyzed by item (Table 2), the items obtaining the lowest scores were those in the “new relational model” dimension, such as “They help me become informed via internet”, “I use internet and my mobile to consult my clinical record” and “I am encouraged to talk to other patients”, all of which were below 3 points, indicating that only 8% of patients always or almost always used the internet to consult their medical history, citations or other data from their health service. 

Only 15% responded that health professionals always or almost always informed them of forums or other reliable websites to obtain information about their disease.

### 3.4. Relationship between Experience and Comorbidity, Adherence and Degree of Control

In the bivariate analysis, we found no association between any of the cardiovascular comorbidities, depression, osteoarthritis and chronic obstructive pulmonary disease and adherence. None of the IEXPAC scores showed significant differences between those with or without comorbidities (Table S2).

In general, noncontrolled patients with respect to their blood glucose, blood pressure or global factors showed numerically higher IEXPAC scores; however, the differences were significant only in the total score for patients without adequate glycemic control according to guideline recommendations and in the total score and self-management for patients who did not have adequate control of all three factors (Table 3).

Compliant patients, either individually with oral antidiabetics, antihypertensive drugs and lipid-lowering agents or globally with all three drug categories, showed a better experience (numerically) than nonadherent patients, although the differences were significant only in the total score and productive relationships in patients adherent to lipid-lowering agents compared to nonadherent patients (Table 4).

## 4. Discussion

This study demonstrates that patients with diabetes and comorbidities such as hypertension and dyslipidemia exhibit a limited satisfactory experience with healthcare, with several areas requiring important improvement, especially the use of new technologies in their relationship to their healthcare and disease management, fostering contact with other patients, and, to a lesser extent, providing information on other health and social resources of interest to patients. For those patients requiring hospitalization, there is also significant room for improvement in the continuity of healthcare.

The IEXPAC dimension most affected in terms of worse experience was the “new relational model”, which includes promoting the proper use of new technologies to learn about their disease or contact health services and fostering relationships with other patients. In the analysis of this dimension by age groups we found that the score worsened as the age increased. Although it is expected that elderly patients will be less willing to use new technologies, there are studies showing that even this age group may benefit and adapt fast to eHealth [19,20,21]. This encouragement occurs with a high frequency (always or almost always) in less than one-sixth of patients in the case of new technologies and in approximately a quarter of patients with regard to fostering contact with other patients. These results are consistent with those reported in a previous study using IEXPAC in patients with diabetes and cardiovascular or renal disease and with those observed in that same study for other chronic diseases such as rheumatic diseases, inflammatory bowel disease or HIV infection [17,22]. This may suggest that this is an area of improvement in the patient experience that depends not on the type of chronic disease but on healthcare. These results also reinforce the importance of seeking a triple goal [23] in healthcare focused on improving the patient experience and population health (specially through the improvement of the relationship between health care professionals and patients) [24,25], reducing the per capita cost of human-centered care, optimizing resources and making the use of new technologies essential. A recent publication about the healthcare experience of Spanish patients with T2DM also highlights the importance of a good healthcare professional-patient relationship, based on emotional links and interpersonal trust, with regular follow-up by the same physician and involvement of nursing to improve diabetes management [26].

However, there are some disease-related differences that the previous study with IEXPAC shows, which can help to improve these areas. Although the promotion of the use of the internet to learn about their disease is low in all therapeutic areas, it is lower in those areas involving older patients (i.e., those with diabetes and rheumatic diseases), suggesting that age can be considered a barrier to the promotion of these technologies, an aspect that particularly affects patients with type 2 diabetes. The second aspect, highlighted by the authors of the work themselves, is that HIV patients had a better situation in all areas than the rest of the patients. As the authors of the work suggest, this may be due to the prioritization and investment in resources that were made over several years for HIV patients [17], suggesting that it is possible to improve these aspects by investing in the programs needed to do so.

The use of new technologies could improve the patient experience. A study in public hospitals in China showed that patients using mobile health apps had a better experience, especially with regard to access to health information, and better short-term health outcomes [27]. The ValCrónic study [21] consisted of a five-year telemonitoring program in four Spanish healthcare centers that included 500 high-risk patients with diabetes, elevated blood pressure (BP), heart failure or chronic obstructive pulmonary disease. The use of telemedicine in this study had a significant impact on in the control of weight, heart rate, systolic and diastolic BP and HbA1c. In addition, a significant reduction in the percentage of people who needed emergency services was observed. It is important to note that according to some studies, only a relatively small proportion of healthcare professionals, doctors and nurses believe that patient involvement with their healthcare improves health outcomes [28]. In our view, this suggests the need for programs that increase the knowledge of the barriers that hinder the implementation of these new technologies in the management of these patients and facilitate their incorporation by healthcare professionals. Especially at the present time, the COVID-19 pandemic and its social confinement-related consequences are an additional challenge for the proper management of T2DM patients [29]. In this sense, the use of new technologies, mainly telemedicine, is a very interesting solution, as they allow rationalization and optimization of the resources available (especially in times of crisis), facilitating contact between health professionals and patients and promoting self-care and the exchange of key information for the diagnosis and management of chronic diseases. The “Telemedicine Revolution” has to occur for quality medical care [30]. With this change in the relationship with the patient, which helps ensure continuity of care, it is essential to evaluate patients’ experiences with the healthcare system before, during and after implementing the new care model.

Another aspect indicated by our results and those of the previous study conducted in Spain is the relatively poor experience of patients with the healthcare they receive during follow-up after hospital discharge. In Spain, these results are also consistent with those obtained in other areas, such as rehabilitation, in which a frequent disconnection between the programs received in the hospital and those subsequently received has been reported [31]. Moreover, a qualitative study conducted in four Australian hospitals showed that patients considered that good communication and quality information were important both at hospital admission and discharge and that follow-up at home was a key element [32]. A study of patients requiring continued follow-up care conducted in Spain also noted the importance of improving patient information and education at the time of hospital discharge. Notably, almost one-third of patients in this study had doubts about their health status and the management of their disease just 24 h after discharge [33].

With regard to the association between experience and disease control, our results show an inverse association, i.e., the lack of control in some cases (e.g., HbA1c control with global experience) seems to be associated with a better experience with health professionals and services. There are similar studies performed also in other countries about the perception of cardiovascular risk factors in physicians and patients although they were using different methodology [34,35,36,37,38,39]. Differences in this regard seem more consistent with those who do not have all three risk factors under control. Although this is a cross-sectional study, the hypothesis that those patients with the worst disease control receive greater attention from healthcare professionals, resulting in an improvement in the patient’s perception of the health experience, could be established. It could be possible that people with poor disease control need more appointments to reduce HbA1c levels or that healthcare professionals make more follow-up visits with those patients. Moreover, in a study looking for the influence of frequency of medical visits and HbA1c control, improvement in HbA1c control was not related to a higher number of visits to the doctor but to treatment intensification [40]. Unfortunately, information on this topic is scanty. We also found, especially in the case of lipid-lowering agents, an association between experience and better adherence. This result is consistent with what is reported in the literature, as the positive association between experience and adherence has been most consistently studied and demonstrated in previous studies [14]. Since this is a cross-sectional study, causality cannot be inferred, but the hypothesis that a good experience with the system favors adherence seems more plausible than the opposite.

This study has several limitations. Due to the aforementioned cross-sectional nature, we need to consider the representativeness of our sample, since we included patients with diabetes and other cardiovascular risk factors such as hypertension and dyslipidemia. Although the presence of these comorbidities is very common in patients with diabetes, approximately 45% of T2DM patients in Spain also have hypertension and dyslipidemia concomitantly, while another 42% have either hypertension or dyslipidemia (results from a recent study) [10]. In addition, although the sample size was appropriate for determining the study’s main objectives, it could be interesting to have a higher number of patients in a subsequent study, which would allow subgroups (e.g., based on age ranges) to be identified where clearer associations among adherence, metabolic control and patient experience could be established.

## 5. Conclusions

Our study identifies the existence of a wide margin for improvement in the experience of T2DM patients with the healthcare system, especially regarding the information that patients receive or can obtain—importantly, the use of new technologies for these purposes—and, for those requiring hospitalization, regarding their follow-up after hospital discharge.

## Figures and Tables

**Table 1:** Baseline characteristics.

Variable	IEXPAC Population (*n* = 248)	IEXPAC 11 Items (*n* = 229)	IEXPAC Item No. 12 (*n* = 124)
Age (years), mean (SD)	70.9 (9.17)	70.7 (9.29)	70.3 (9.33)
Woman, *n* (%)	119 (48.0)	111 (48.5)	52 (41.9)
Caucasian, *n* (%)	247 (99.6)	228 (99.6%)	123 (99.2)
Weight (kg), mean (SD)	81.4 (14.1)		
BMI (kg/m^2^), mean (SD)	31.3 (4.9)		
Patient living with others, *n* (%)	221 (89.1)	204 (89.9)	113 (92.6)
**Risk factors, mean (SD)**			
HbA1c (%)	6.73 (0.98)	6.74 (0.99)	6.87 (1.05)
LDLc	85.95 (27.67)	85.93 (28.1)	86.59 (28.38)
SBP (mm/Hg)	133.01 (13.65)	132.51 (13.81)	133.93 (14.01)
DBP (mm/Hg)	75.63 (8.96)	75.42 (8.98)	75.98 (9.07)
Mean no. of risk factors according to therapeutic guidelines (SD)	2.00 (0.83)		
Mean No. of controlled risk factors based on clinical opinion (SD)	2.43 (0.70)		
**Treatments, mean (SD)**			
No. daily OAD	2.62 (1.79)	2.67 (1.84)	2.63 (1.92)
No. daily AHT	1.77 (0.96)	1.77 (0.98)	1.79 (1.06)
No. daily LLD	1.16 (0.46)	1.17 (0.47)	1.15 (0.46)
No. daily OAD, AHT and LLD	5.55 (2.26)	5.60 (2.31)	5.58 (2.50)
Mean No. of concomitant tablets (other than OAD, AHT and LLD) (SD)	4.19 (3.74)	4.23 (3.78)	4.51 (4.02)
Mean No. of concomitant tablets (OAD, AHT, LLD + concomitant drugs) (SD)	9.74 (4.40)	9.83 (4.44)	10.09 (4.70)
**Diabetes-related complications, *n* (%)**			
Retinopathy	15 (6.3)	15 (6.8)	11 (9.1)
Nephropathy	38 (15.6)	35 (15.6)	28 (22.8)
Neuropathy	6 (2.5)	5 (2.2)	3 (2.4)
Diabetic foot	1 (0.4)	1 (0.4)	0
**Comorbidities, *n* (%)**			
Coronary heart disease	50 (20.2)	47 (20.5)	28 (22.5)
Heart failure	16 (6.5)	15 (6.6)	10 (8.1)
Occlusive peripheral artery disease	13 (5.2)	12 (5.3)	5 (4.1)
Cerebrovascular disease	21 (8.5)	21 (9.2)	10 (8.1)
Depression	40 (16.2)	38 (16.7)	18 (14.6)
Arthrosis	107 (43.7)	99 (43.8)	55 (45.1)
Chronic obstructive pulmonary disease	20 (8.1)	17 (7.5)	11 (9.0)

OAD, oral antidiabetics; AHT, antihypertensive drugs; SD, standard deviation; LDLc, low-density lipoprotein cholesterol; LLD, lipid-lowering drugs; BMI, body mass index; SBP, systolic blood pressure; DBP, diastolic blood pressure.

**Table 2:** Patient experience with healthcare professionals and care: IEXPAC results by item.

Dimension		Item	Mean Score (From 1—Never to 5—Always)	% “Always” or “Almost Always”
**Dimension 1** **Productive interactions**	1	They respect my lifestyle	4.6	91.9
	2	They are coordinated in offering me good care	4.5	87.0
	5	They ask me about and help me follow my treatment plan	4.7	94.3
	9	They are concerned about my wellbeing	4.6	94.3
**Dimension 2** **New relational model**	3	They help me become informed via the internet	2.0	15.4
	7	I use the internet and my mobile phone to consult my clinical record	1.5	8.1
	11	They encourage me to talk with other patients	2.6	27.4
**Dimension 3** **Patient’s self-care**	4	Now I know how to look after myself better	4.3	82.4
	6	We agree on objectives to have a healthy life and to control my health problems better	4.6	89.5
	8	They ensure I take my medication correctly	4.6	91.5
	10	They inform me about health and social resources that can help me	3.6	59.4
			**Mean score (from 0—never to 10—always)**	**% “always” or** **“almost always”**
**Item 12** **Continued care after hospital discharge**	12	They care about me upon my arrival home after being in hospital	6.2	57.3

IEXPAC, Instrument for the Evaluation of the Experience of Chronic Patients. IEXPAC mean overall score, Item 12 mean score, and mean scores for the three dimensions were also assessed stratified by age group: <65 years; 65 to 70 years; 70 to <80 years and >80 years. There were no statistically significant differences in these scores by age group except for the dimension “new relational model” that showed a better score at lower ages (Table S1).

**Table 3:** Patient experience and his/her relationship with the degree of control.

IEXPAC Score	HbA1c		LDLc		Blood Pressure		All Factors	
	Control	No Control	Control	No Control	Control	No Control	Control	No Control
Total	6.8	7.1 *	6.9	6.9	6.8	7.0	6.6	7.0 *
Productive relationships	8.9	9.2	9.0	8.9	8.9	9.1	8.7	9.1
New relational model	2.4	2.8	2.4	2.7	2.6	2.3	2.0	2.7 *
Self-management	8.1	8.5	8.2	8.1	8.0	8.4	7.9	8.3 *

LDLc, Low-density lipoprotein cholesterol; IEXPAC, The Instrument for the Evaluation of the Experience of Chronic Patients. All figures are mean values; * *p* <0.05.

**Table 4:** Patient experience and treatment adherence.

IEXPAC Score	OAD		AHT		LLD		Total	
	Adherent	Non-Adherent	Adherent	Non-Adherent	Adherent	Non-Adherent	Adherent	Non-Adherent
Total	6.9	6.8	6.9	6.7	7.0 *	6.4	7.0	6.6
Productive relationships	9.0	9.0	9.0	8.9	9.1 **	8.4	9.0	8.8
New relational model	2.5	2.4	2.5	2.1	2.6	2.1	2.6	2.3
Self-management	8.2	8.0	8.2	8.1	8.2	7.7	8.3	7.9

OAD, oral antidiabetics; AHT, antihypertensive drugs; LLD, lipid-lowering drugs; IEXPAC, Instrument for the Evaluation of the Experience of Chronic Patients. All figures are mean values; * *p* <0.05; ** *p* < 0.01.

## Data Availability

The data that support the findings of this study are available from the corresponding author upon reasonable request.

## Supplementary Materials

The following are available online at https://www.mdpi.com/article/10.3390/jcm10132831/s1, Table S1: Results of the IEXPAC questionnaire stratified by age group. Table S2: Patient experience with healthcare professionals and care based on the presence of comorbidities.
