# Supplementary material for: Experience of Patients with Diabetes and Other Cardiovascular Risk Factors with Health Professionals and Healthcare in Spain
Source: J Clin Med. 2021 Jun 26;10(13):2831. doi: 10.3390/jcm10132831 (PMC8267612; doi:10.3390/jcm10132831)
Supplement: Supplementary file 1 [file jcm-10-02831-s001.zip › jcm-1231511-supplementary.pdf]

Table S1. Results of the IEXPAC questionnaire stratified by age group

Age group (years)	IEXPAC overall score			Item 12			Productive interactions			New relational model			Patient self-management		
	**N**	**Mean value (SD)**	**p-value**	**N**	**Mean value (SD)**	**p-value**	**N**	**Mean value (SD)**	**p-value**	**N**	**Mean value (SD)**	**p-value**	**N**	**Mean value (SD)**	**p-value**
< 65	47	7.13 (1.37)	0.155	25	5.80 (4.43)	0.061	47	8.91 (1.56)	0.764	48	3.12 (2.55)	0.005	47	8.35 (1.54)	0.315
65 to <70	49	7.12 (1.60)		29	7.07 (4.28)		53	8.83 (1.37)		50	3.20 (2.95)		54	8.43 (1.51)	
70 to <80	91	6.70 (1.20)		91	5.19 (4.64)		96	9.01 (1.24)		94	2.06 (2.04)		96	8.02 (1.68)	
> 80	42	6.71 (1.39)		42	8.056 (2.91)		47	9.10 (1.27)		42	1.96 (2.50)		46	8.00 (1.50)	

IEXPAC, Instrument for the Evaluation of the Experience of Chronic Patients.

Table S2. Patient experience with healthcare professionals and care based on the presence of comorbidities.

	IEXPAC score				
**Comorbidity**	**Total**	**Productive interactions**	**New relational model**	**Self-management**	**Follow-up after hospital discharge**
With coronary artery disease	7.0	6.2	9.1	2.8	8.1
Without coronary artery disease	6.9	6.2	8.9	2.5	8.2
With heart failure	6.6	7.3	9.0	1.9	7.7
Without heart failure	6.9	6.1	9.0	2.6	8.2
With peripheral artery disease	6.9	5.5	9.1	2.2	8.3
Without peripheral artery disease	6.9	6.3	9.0	2.5	8.2
With cerebrovascular disease	6.8	6.3	9.1	1.7	8.2
Without cerebrovascular disease	6.9	6.2	9.0	2.6	8.2
With depression	6.7	5.8	8.8	2.4	8.0
Without depression	6.9	6.3	9.0	2.5	8.2
With arthrosis	6.7	5.9	8.9	2.2	8.0
Without arthrosis	7.0	6.4	9.0	2.8	8.3
With COPD	6.5	7.0	8.9	2.2	7.8
Without COPD	6.9	6.2	9.0	2.5	8.2

IEXPAC, Instrument for the Evaluation of the Experience of Chronic Patients.
